# A Review on the State of the Art in Copter Drones and Flight Control Systems

**DOI:** 10.3390/s24113349

**Published:** 2024-05-23

**Authors:** Janis Peksa, Dmytro Mamchur

**Affiliations:** 1 Information Technologies Department, Turiba University, Graudu Street 68, LV-1058 Riga, Latvia; janis.peksa@turiba.lv; 2 Institute of Information Technology, Riga Technical University, Kalku Street 1, LV-1658 Riga, Latvia; 3 Computer Engineering and Electronics Department, Kremenchuk Mykhailo Ostrohradskyi National University, Universitetska Street 20, 39600 Kremenchuk, Ukraine

**Keywords:** UAV, multirotor drones, flight controller, classification, copters

## Abstract

This paper presents an overview on the state of the art in copter drones and their components. It starts by providing an introduction to unmanned aerial vehicles in general, describing their main types, and then shifts its focus mostly to multirotor drones as the most attractive for individual and research use. This paper analyzes various multirotor drone types, their construction, typical areas of implementation, and technology used underneath their construction. Finally, it looks at current challenges and future directions in drone system development, emerging technologies, and future research topics in the area. This paper concludes by highlighting some key challenges that need to be addressed before widespread adoption of drone technologies in everyday life can occur. By summarizing an up-to-date survey on the state of the art in copter drone technology, this paper will provide valuable insights into where this field is heading in terms of progress and innovation.

## 1. Introduction

In recent years, copter drones have experienced a remarkable surge in popularity, driven by advancements in technology and their versatile applications across various industries [1,2,3,4,5]. Originally developed for military purposes, these unmanned aerial vehicles (UAVs) have transitioned into civilian domains, revolutionizing fields such as aerial photography, surveillance, agriculture, and search and rescue. The ability of copter drones to navigate and hover in confined spaces, coupled with advancements in miniaturized sensors and efficient propulsion systems, has made them indispensable tools for tasks that were once impractical or costly. This pervasive integration of copter drones across sectors highlights their transformative impact on industries and underscores the need for a comprehensive understanding of their state-of-the-art capabilities and flight control systems.

Understanding the current state of the art in copter drones and flight control systems holds significant importance for several reasons.

Technological Advancements. The field of copter drones is rapidly evolving with continuous technological advancements. Staying abreast of the latest developments allows researchers, engineers, and practitioners to leverage cutting-edge technologies for enhanced drone performance, safety, and efficiency [6,7].

Safety and Regulations. As copter drones become more ubiquitous, ensuring their safe integration into airspace is crucial. Knowledge of the state of the art in flight control systems aids in designing robust safety mechanisms, meeting regulatory requirements, and mitigating potential risks associated with drone operations [8,9].

Optimizing Performance. Understanding the latest advancements in flight control systems enables the optimization of drone performance. This includes improvements in stability, maneuverability, and responsiveness, contributing to better outcomes in various applications such as aerial surveillance, package delivery, and agriculture [10,11].

Applications Across Industries. Copter drones find applications in diverse industries, including agriculture, cinematography, infrastructure inspection, and disaster response. A comprehensive understanding of the state-of-the-art technology allows professionals in these sectors to harness the full potential of drones for improved efficiency, cost-effectiveness, and data collection.

Innovation and Research. Researchers and academics benefit from knowledge about the current state of the art to identify gaps in existing technologies, propose novel solutions, and contribute to the ongoing innovation in drone technology. This understanding serves as a foundation for further research and development in the field [12,13].

Market Dynamics. For businesses involved in the production, sale, or service of copter drones, awareness of the current state of the art is essential for strategic decision making. This knowledge aids in identifying market trends, customer demands, and potential areas for investment or collaboration.

Addressing Challenges. The drone industry faces challenges such as battery life limitations, regulatory hurdles, and public perception [14]. A thorough understanding of the current state of the art allows stakeholders to address these challenges effectively, driving progress and acceptance of drone technology.

The proposed work aimed to analyze current advances in copter drones, paying attention to indicated importance fields and helping readers to properly choose the topical direction of their research work. Staying informed about the current situation in the copter drone industry is fundamental for unlocking their full potential, ensuring safe integration into society, and driving continuous innovation across various sectors.

This manuscript is organized as follows. Section 2 discusses unmanned aerial vehicle types and different types of their classification, focusing mostly on multicopters as the most affordable and massively used, concluding with a brief discussion on unmanned vehicle complexes and their main tasks. Section 3 provides a detailed discussion on multirotor drone types, and their differences and pros and cons, summarizing findings in a table view. Section 4 is devoted to a discussion on typical applications where copter drones could currently find themselves. Section 5 discusses typical methods used to control drones, including traditional and advanced. Section 6 surveys typical sensor types used to ensure drone operability and their purposes. Section 7 discusses the most popular software used for drone operation and drone control. Finally, Section 8 highlights current challenges faced by unmanned aerial vehicles and their implementation and possible directions for advances in this area.

## 2. Types of Unmanned Aerial Vehicles

**Figure 1:**
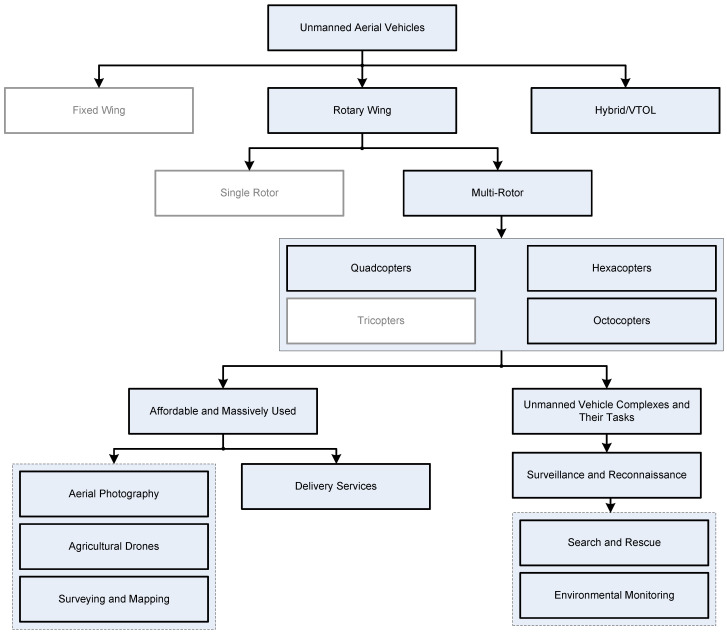
Section 2 discussion structure.

This section provides UAV classification followed by a discussion on the main types and configurations of UAVs, focusing on the most affordable and massively used. The structure of the discussion in this section is presented in a block diagram (Figure 1). The central block represents the main topic: UAV types. Next-level blocks represent the UAVs’ categories basing on their structural and operational characteristics. In the current work, the main attention is focused on rotary wing UAVs and there is little discussion on VTOL drones. Next-level subcategories discuss typical tasks and applications of rotary UAVs and UAV complexes.

Topics that are not discussed within this section are presented in white rectangles with grey-colored text and borders.

### 2.1. Classifications of Unmanned Aerial Vehicles

An unmanned aerial vehicle is an aircraft designed to fly without a pilot on board, the flight control and steering of which being carried out by an appropriate program or with the help of a special control station located behind the aircraft [1,15].

An unmanned aircraft complex (unmanned aircraft system) includes an unmanned aircraft, remote piloting points (ground control stations), necessary control and control lines, and other elements specified in the approved design of the type of this complex [16]. This complex may include several unmanned aerial vehicles. In other words, a UAV is an aircraft controlled by one or more pilots using communication channels.

UAVs can be classified by [1,14,15,16,17,18]:

- Scope of tasks (purpose);
- Power system (drive type);
- Aircraft type (design);
- Flight duration;
- Control system type;
- Mass;
- Wing type;
- Flight height;
- Base type;
- Type of fuel tank;
- Radius of action;
- Maximum flight speed;
- The number of engines;
- Take-off/landing type;
- The time of receiving the collected information.

Selected classifications are provided below.

According to the purpose, the following are distinguished [1,16]:

- Commercial UAVs used for profit, in particular, in agriculture, video recording, geological research, etc.;
- Military UAVs designed for military operations, reconnaissance, support, communication tasks, etc.;
- Civilian UAVs used for civilian purposes, such as search and rescue, environmental monitoring, scientific research, etc.

UAVs can also be classified by drive type [15,16]:

- Electric UAVs—need an electric power source for flight;
- Hybrid UAVs—use both electric and fuel as the power system;
- Fuel UAVs—driven by an internal combustion engine.

This classification is used to determine the types and characteristics of UAVs depending on the fields of their use and functional capabilities.

According to the design, UAVs are divided into five main categories [1,15,16,17].

(1) Aircraft (fixed-wing), including the following:
  - Monoplanes—one-wing construction;
  - Biplanes—use two wings—upper and lower;
  - Triplanes—use three wings located one above the other;
  - Wings—delta-shaped construction.
(2) Multirotor UAVs, which include the following:
  - Quadcopters, four rotors;
  - Hexacopters, six rotors;
  - Gyrocopters (octocopters), eight rotors.
(3) Tailsitters—a combination of fixed wings and multirotors that uses the advantages of both designs.
(4) VTOL (vertical take-off and landing)—includes UAVs that can perform vertical take-off and landing and then operate in horizontal flight mode.
(5) Balloons and airships—ultralight vehicles that operate using forces of air and can have a gas cylinder for lifting.

According to the range and duration of the flight, UAVs are characterized as follows [15,17]:

- Short-endurance—with a flight range and duration of up to an hour;
- Medium-endurance—from one to several hours;
- Long-endurance UAVs—the range and flight duration of which are more than several hours (up to several dozen hours).

The classification of the International Association for Unmanned Vehicle Systems, UVSI, distinguishes UAVs according to the following [19,20,21]:

- Flight mass;
- Flight duration;
- Flight endurance;
- Flight altitude;
- Areas of use.

This classification is summarized in Table 1.

Each of these structures has its own advantages and limitations but, in this research, we are focused mostly on multirotor UAVs as one of the most popular and easy-to-use vehicles for personal and civilian purposes.

### 2.2. Multicopters

**Figure 2:**
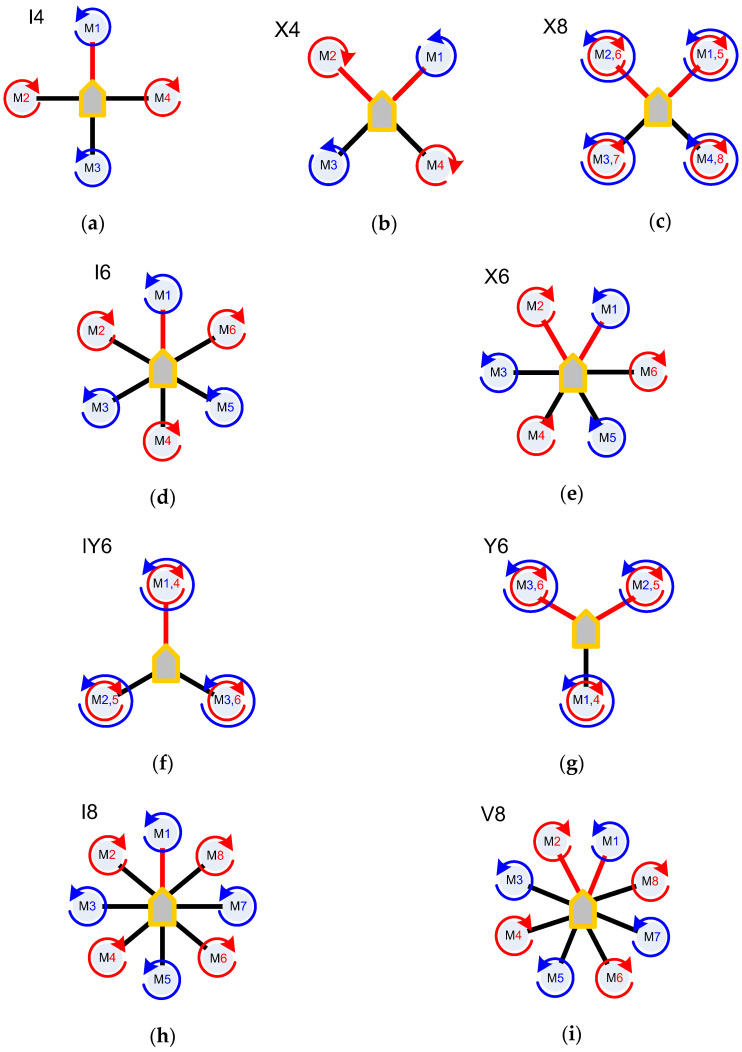
Symmetrical multicopter designs: (**a**) I4—quadcopter “+”; (**b**) X4—quadcopter “x”; (**c**) X8—coaxial octocopter; (**d**) I6—hexacopter “+”; (**e**) X6—hexacopter “x”; (**f**) IY6—coaxial hexacopter “+”; (**g**) Y6—coaxial hexacopter “x”; (**h**) I8—octocopter “+”; (**i**) V8—octocopter “x”.

This paper is focused on the most popular UAVs for civil use, namely, multicopters or multirotors. These include UAVs with more than two propellers. Reactive moments are balanced due to the rotation of the support propellers in pairs in different directions or the inclination of the thrust vector of each propeller in a certain direction. Unmanned multicopters are usually mini and micro UAVs. The most common design of multicopters is a quadcopter. The presence of four rigidly fixed rotors makes it quite easy to lift this UAV into the air. In total, nine symmetrical designs of multicopters are known [22,23], which differ in the location of the frame and the number of support propellers [24,25] (Figure 2). The frame consists of a platform for power and control units and shoulders formed by beams.

Conventional designation of the scheme is formed using an alphabet character indicating the frame shape and a numerical character indicating the number of motors. The upright frame shape is designated by the characters “I” and “+” (plus) and the cross-shaped frame is designated by the characters “V”, “X”, and “Y”. For example, the IY6 is an upright frame hexacopter with three beams. If one of the beams coincides with the longitudinal axis of the multicopter, then the frame shape is considered to be upright. If the longitudinal axis divides the angle between two adjacent beams in half, then the frame shape is considered to be cross-shaped. The number of support propellers depends on the total thrust required for flight. To compensate for the reactive torque, they are placed opposite each other or in a coaxial scheme. The Y4 quadcopter provides a more stable operation compared to the tricopter. The motor efficiency increases by one-third. The X4 [26] and I4 [27] quadcopters are equipped with four support propellers located at the ends of the frame beams. This is the simplest design of a four-propeller multirotor. There is no separate yaw control mechanism, which makes the flight less stable. At the same time, with such a design, the motors work most efficiently. Another advantage of the X4 and I4 quadcopter is the ability to programmatically select the frame shape. The design of the VTail quadcopter is similar to both a tricopter and a Y4 quadcopter: typically, two propellers are located at the ends of the beams in front [28]. The other two, which are used to control the yaw, are next to each other in the tail section at a certain angle to the frame. Compared to an X4 or I4 quadcopter, VTail motors require more power, but the yaw control is more predictable. The design of Y6 and IY6 hexacopters is similar to the design of tricopters [29]. However, instead of a servo for yaw control, a coaxial arrangement of support propellers is used. The advantage of such multicopters over I6 and X6 hexacopters is its compactness on the one hand. On the other hand, the coaxial arrangement of the support propellers leads to a decrease in the motor’s efficiency [30]. The design of the Y6 and IY6 hexacopters also allows for software selection of the frame orientation.

A pentacopter is an asymmetric multicopter scheme [31]. The frame of such an aircraft has five beams: the front two at an angle of 120° between them and the other three at an angle of 60° between them. This design is similar to the I6 hexacopter without the front beam. The obvious advantage of the pentacopter is a large angle between the front beams, which eliminates the problem of parts of the multicopter structure falling into the frame on one shoulder. The V8 octocopter performs controlled flight when four motors on two adjacent arms fail [32]. Due to this, the safety and stability of the flight is better than those of hexa-, quad-, and tricopters. However, I8 and V8 octocopters require significant energy consumption. The X8 octocopter has eight propellers arranged in a coaxial pattern [33]. The design of such a multirotor is similar to the design of a quadcopter. The X8 octocopter has better controllability thanks to the ability to perform yaw control. However, due to the coaxial design, the motors are used less efficiently than in I8 and V8 octocopters.

Also, it should be mentioned that the main features of a copter design could be found in a fixed-wing UAV with a vertical take-off and landing (VTOL) system [34,35]. These UAVs have four or more propellers for take-off and landing and fixed wings for long-range flight.

Peculiarities of the most widely used copter designs are discussed in Section 3.

### 2.3. UAV Complex

The UAV complex contains three key components that work together to effectively perform tasks [36,37,38]:

1. The UAV itself. It is an aircraft or drone that carries out missions in the air. It is equipped with various sensors, cameras, and other devices for collecting information.

2. The ground station or ground base, which is the place where the drone is launched and lands, as well as where the equipment for communication and control over it is installed. A ground station can be mobile (such as a vehicle or ship) or stationary (such as a land base). The ground station contains additional equipment necessary for the operation of the UAV, the antenna system, and the catapult.

3. The operator, who is a person or a team of people who are responsible for operating and controlling the UAV during the flight [39,40,41,42]. The operator interacts with the drone through a ground station using a remote control or a computer used to send commands and receive information from the drone’s sensors and cameras.

Among the main functions of the operator, there are the following:

- Mission planning;
- Setting flight parameters;
- Data monitoring from sensors and cameras;
- Decision making in accordance with the received data.

The UAV operator is responsible for planning missions, including determining flight paths, waypoints, and operational parameters [43]. Mission planning involves considering factors such as weather conditions, airspace regulations, and mission objectives.

Also, the operator controls the UAV’s navigation and flight parameters, including steering, altitude control, and speed adjustments [40,43]. Depending on the mission requirements, the operator may manually pilot the UAV or utilize autonomous flight modes for waypoint navigation and autonomous operations.

In the event of emergencies or unexpected situations, the operator takes appropriate actions to mitigate risks and ensure the safety of the UAV and surrounding environment [40,43]. This may involve initiating emergency procedures, executing a safe landing, or implementing contingency plans as needed.

## 3. Types of Multirotor Drones

**Figure 3:**
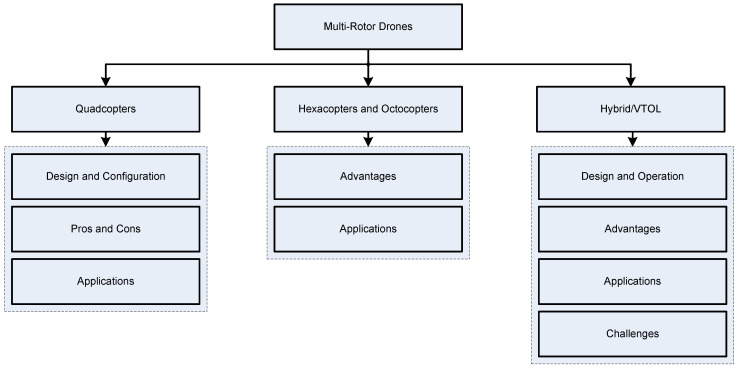
Section 3 structure.

This section discusses peculiarities and main applications of the most widely spread multirotor drone types, namely, quadcopters, hexacopters, octocopters, and VTOL drones. The section structure is outlined in Figure 3.

### 3.1. Quadcopters

A quadcopter, also known as a quadrotor, is a type of copter drone that features four rotors. Each rotor is mounted at the end of a horizontal arm, and the propellers generate lift by spinning in opposite directions. Quadcopters are widely popular due to their simple design, stability, and ease of control. The main features of quadcopters and their applications are listed below [44,45,46,47,48,49,50,51,52].

**Figure 4:**
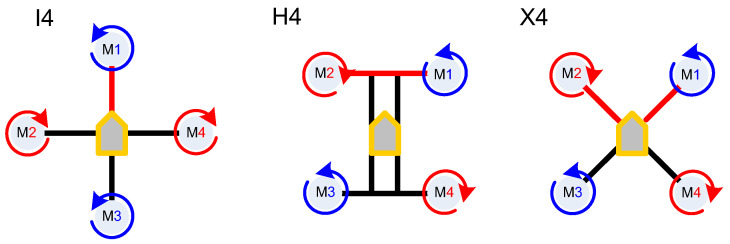
Quadcopter designs: X-shaped (I4 and X4), H-shaped (H4).

Design and Configuration. Quadcopters have a symmetrical X- or H-shaped or hybrid frame with four arms, each supporting a rotor [44,45,46,47,53] (Figure 4).

The rotors are arranged in a square or rectangular pattern, and they can be independently controlled for stability and maneuverability. The drone’s flight is controlled by adjusting the speed of each rotor, allowing it to ascend, descend, rotate, and move horizontally.

Stability and Maneuverability. Quadcopters are known for their inherent stability, achieved through the balancing of opposing torques generated by pairs of rotors [54]. This design allows for precise and agile movements, making quadcopters suitable for various applications that require controlled and stable flight.

Applications. As one of the cheapest and most stable multicopter solutions, quadcopters are found in various applications, as listed below [44,45,46].

Aerial Photography and Videography. Quadcopters are extensively used for capturing high-quality images and videos from unique vantage points. They have become popular tools for filmmakers, photographers, and content creators.

Surveillance and Security. Quadcopters equipped with cameras and sensors are employed for surveillance purposes, providing a cost-effective and efficient way to monitor large areas, borders, and critical infrastructure.

Mapping and Surveying. Quadcopters equipped with specialized sensors, such as LiDAR or multispectral cameras, are used for mapping and surveying terrain, agricultural fields, and construction sites.

Search and Rescue. Due to their agility and ability to access hard-to-reach areas, quadcopters are employed in search and rescue operations. They can quickly survey disaster-stricken areas and relay crucial information to responders.

Recreational Use. Quadcopters are popular among hobbyists for recreational flying. Entry-level models are widely available for users to experience the thrill of piloting a drone.

Educational and Research Purposes. Quadcopters serve as educational tools for teaching principles of aerodynamics, robotics, and programming [51]. Researchers utilize quadcopters for experimentation in fields such as swarm robotics, autonomous navigation, and artificial intelligence. In summary, quadcopters are versatile and widely adopted due to their stability, maneuverability, and diverse range of applications, making them a prevalent choice for both professional and recreational drone users.

### 3.2. Hexacopters and Octocopters

Hexacopters and octocopters are variations of multirotor drones with six and eight rotors, respectively. These configurations offer several advantages over quadcopters, primarily related to increased stability, payload capacity, and redundancy. What follows is an exploration of the use and advantages of drones with multiple rotors [55,56,57,58,59,60,61,62,63].

Enhanced Stability. Hexacopters and octocopters provide increased stability compared to quadcopters [56,57,61]. The additional rotors contribute to a more balanced and controlled flight, especially in challenging weather conditions.

Payload Capacity. The added rotors allow for a higher payload capacity [59]. This makes hexacopters and octocopters suitable for carrying heavier cameras, sensors, or equipment, expanding their applications in professional photography, cinematography, and industrial inspections.

Redundancy and Safety. Hexacopters and octocopters offer redundancy in case of a rotor failure [61,62]. If one or more rotors malfunction, the remaining rotors can compensate, enabling the drone to maintain stability and land safely. This redundancy is particularly crucial in critical missions and applications where reliability is paramount.

Versatility in Applications. As well as quadcopters, hexa- and octocopters might be found in a variety of applications where increased reliability and operability are needed. Selected areas of application are listed below [59,61,62,63,64].

Aerial Photography and Cinematography. Hexacopters and octocopters are preferred in the film and photography industry for capturing high-quality aerial shots. The increased stability allows for smoother footage even in windy conditions.

Industrial Inspections. These drones are used for inspecting infrastructure such as power lines, bridges, and pipelines. Their ability to carry heavier sensors or cameras allows for more advanced inspection capabilities.

Agriculture. Hexacopters and octocopters equipped with specialized sensors can be employed for precision agriculture, monitoring crop health, and optimizing farming practices.

Surveying and Mapping. The higher payload capacity makes them suitable for carrying advanced mapping equipment, including LiDAR sensors, for more accurate surveying and mapping applications.

Search and Rescue. In emergency situations, the redundancy and stability of hexacopters and octocopters are advantageous for conducting aerial search and rescue missions.

Extended Flight Endurance. While additional rotors increase power consumption, hexacopters and octocopters can still achieve longer flight times compared to quadcopters [61]. This extended flight time is valuable in applications that require prolonged aerial coverage.

Customization and Flexibility. The modular nature of hexacopter and octocopter designs allows for greater customization [65,66]. Users can adapt these drones for specific applications by adding or modifying payload options, making them versatile tools for various industries.

So, hexacopters and octocopters offer advantages in terms of stability, payload capacity, redundancy, and versatility. These features make them well-suited for a broad range of applications, particularly in industries where precision, reliability, and extended capabilities are crucial.

### 3.3. Fixed-Wing VTOL Drones

Fixed-wing VTOL (vertical take-off and landing) drones represent a hybrid design that combines features of both fixed-wing aircraft and multirotor drones. These drones offer the benefits of efficient forward flight like fixed-wing aircraft and the ability to vertically take off and land similar to multirotors. The main features and advantages of fixed-wing VTOL drones are listed below [34,35,67,68,69].

**Figure 5:**
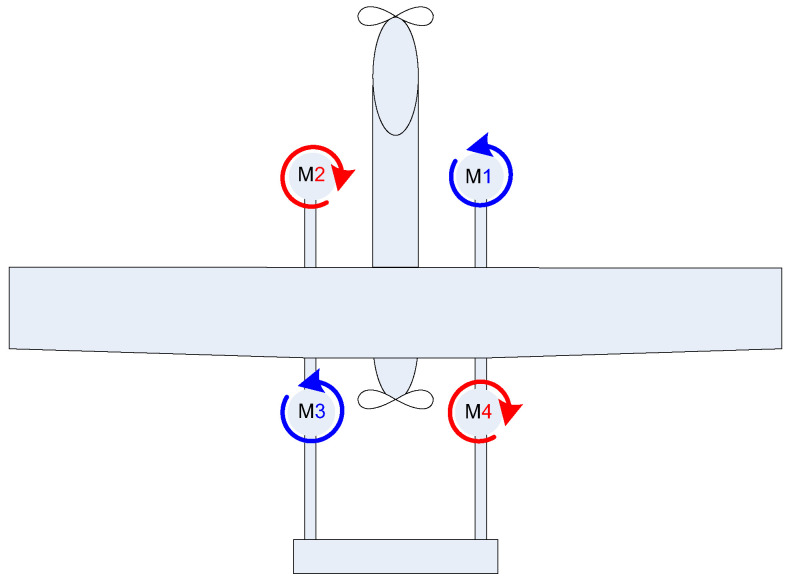
VTOL typical design: motors M1–M4 are used for vertical landing/take-off.

Design and Operation. Fixed-wing VTOL drones typically have a fixed-wing aircraft’s main body and wings, allowing for efficient forward flight [34,67]. They also incorporate rotors or propellers for vertical take-off and landing. During take-off and landing, the rotors are used to provide lift vertically. Once airborne, the drone transitions to forward flight by tilting its rotors or wings. A typical VTOL design is presented in Figure 5.

Advantages. The VTOL design provides certain advantages compared to quad-, hexa-, and octocopters, listed below [34,35,67,70,71].

Efficient Flight. Fixed-wing VTOL drones are more energy-efficient during horizontal flight than traditional multirotors. This efficiency allows them to cover larger distances and stay in the air for extended periods, making them suitable for applications such as mapping, surveying, and surveillance.

Versatility. The ability to take off and land vertically eliminates the need for runways, making fixed-wing VTOL drones versatile in various environments, including urban areas, remote locations, and confined spaces.

Longer Endurance. Compared to purely multirotor drones, fixed-wing VTOL drones can achieve longer flight times. This extended endurance is valuable for applications such as monitoring large agricultural areas or conducting extended surveillance missions.

Payload Capacity. Fixed-wing VTOL drones can carry heavier payloads due to their efficient aerodynamic design. This makes them suitable for tasks that require advanced sensors, cameras, or other specialized equipment.

Applications. Due to the mentioned advantages, VTOLs could be found in the following applications.

Mapping and Surveying. Fixed-wing VTOL drones are well-suited for large-scale mapping and surveying projects [72,73,74]. Their efficiency in covering expansive areas makes them ideal for capturing high-resolution aerial imagery and topographic data.

Agriculture. These drones can be used for precision agriculture, monitoring crop health, and optimizing irrigation [75,76]. The extended flight time and efficiency in covering large fields contribute to their effectiveness in this application.

Infrastructure Inspection. Fixed-wing VTOL drones are employed for inspecting infrastructure such as pipelines, power lines, and bridges [77,78]. Their ability to cover long distances and access remote locations enhances the inspection capabilities in challenging environments.

Emergency Response. The versatility of fixed-wing VTOL drones makes them valuable in emergency response scenarios [79]. They can quickly survey disaster-stricken areas, assess damage, and provide critical information to responders.

Challenges. However, there are certain challenges in VTOL use [70,71,74]. First of all, the transition between vertical and horizontal flight modes introduces complexity to the drone’s design and control systems. Also, fixed-wing VTOL drones can be more expensive than traditional multirotors due to their hybrid design and additional components.

In summary, fixed-wing VTOL drones offer a balance between the efficiency of fixed-wing aircraft and the flexibility of multirotor drones. Their versatility, longer endurance, and payload capacity make them suitable for a wide range of applications across industries.

### 3.4. Comparison of Reviewed Drone Types

A comparison of quadcopters, hexacopters, and octocopters with fixed-wing VTOL drones across various factors is presented in Table 2.

So, each type of copter drone and fixed-wing VTOL drone has its advantages and limitations, making them suitable for different applications and operational requirements. Quadcopters offer agility and ease of use, and hexacopters and octocopters provide increased stability, payload capacity, and endurance, while fixed-wing VTOL drones excel in speed, range, and efficiency for long-distance missions. The choice of drone platform depends on factors such as mission objectives, payload requirements, operating conditions, and budget considerations.

## 4. Applications of Copter Drones

**Figure 6:**
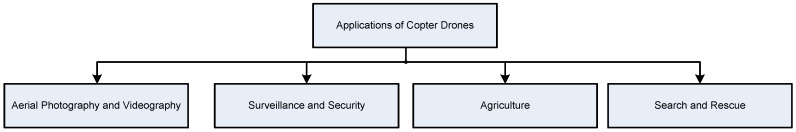
Main applications of rotary drones.

This section discusses the main applications of rotary drones, outlined in Figure 6.

### 4.1. Aerial Photography and Videography

Aerial photography and videography have been revolutionized by the use of drones, enabling the capture of stunning images and videos from unique perspectives. Drones equipped with high-quality cameras and stabilization systems have become invaluable tools for photographers, cinematographers, and content creators. What follows is an examination of how drones are used for capturing high-quality aerial images and videos [80,81,82].

Unique Perspectives. Drones provide a unique vantage point by allowing photographers and videographers to capture images and footage from elevated angles and viewpoints that would be otherwise inaccessible or impractical [80].

Stabilization Systems. Advanced stabilization systems, such as gimbal technology, help drones to maintain steady and smooth shots even in turbulent conditions. This ensures that the captured images and videos are free from unwanted shakes or vibrations [80,81].

Flexibility and Maneuverability. Drones offer unparalleled flexibility in movement, allowing operators to fly smoothly through the air and change perspectives effortlessly [80,81,82,83]. This maneuverability enables creative shots and dynamic compositions that were challenging or impossible to achieve with traditional methods.

Portability and Accessibility. Drones are compact and portable, making them easy to transport to various locations [81]. This accessibility allows photographers and videographers to reach remote or hard-to-access areas, capturing imagery that was once difficult or expensive to obtain.

Real-time Monitoring. Many drones are equipped with live-streaming capabilities, enabling real-time monitoring of the camera feed on a remote controller or mobile device. This feature helps operators to frame shots effectively and make immediate adjustments for optimal results [83].

High-Resolution Cameras. Drones often come equipped with high-resolution cameras, including models capable of shooting in 4K or even higher [82]. This allows for the capture of detailed and visually stunning images and videos suitable for professional use in industries such as filmmaking, advertising, and marketing.

Photogrammetry and Mapping. Drones equipped with specialized cameras and sensors can be used for photogrammetry, creating detailed 3D models and maps of terrain. This application is valuable in fields such as urban planning, construction, and environmental monitoring [82,83,84,85].

Autonomous Flight and Intelligent Features. Some drones feature autonomous flight modes and intelligent features such as waypoint navigation and object tracking [84,85,86]. These capabilities enhance the precision and efficiency of capturing specific shots or following a subject, freeing up the operator to focus on creative aspects.

Time-Lapse and Slow-Motion. Drones allow for the creation of captivating time-lapse sequences and slow-motion footage [87,88]. This flexibility in capturing time and motion adds a dynamic element to the visual storytelling process.

Cost-Effectiveness. Utilizing drones for aerial photography and videography is often more cost-effective compared to traditional methods such as helicopters or airplanes [85]. Drones provide similar capabilities at a fraction of the cost, making aerial imagery more accessible to a broader range of professionals.

In summary, drones have transformed aerial photography and videography by providing a versatile, cost-effective, and accessible platform for capturing high-quality images and videos. The combination of advanced camera technology, stabilization systems, and intelligent features has opened up new possibilities for creative expression and visual storytelling.

### 4.2. Surveillance and Security

Copter drones, particularly multirotor drones, play a significant role in surveillance and security applications, providing a versatile and efficient means of monitoring and securing areas. The use of drones in these contexts has grown rapidly due to advancements in technology, affordability, and their ability to access areas that may be challenging for traditional surveillance methods. What follows is an investigation into the role of copter drones in surveillance and security [89,90,91,92].

Wide-Area Monitoring. Copter drones equipped with high-resolution cameras and sensors are capable of conducting wide-area monitoring [89,90,91,92,93,94]. They can cover large expanses of land, providing real-time aerial views of areas that might be difficult to monitor using ground-based surveillance.

Rapid Deployment. Drones can be quickly deployed to a specific location, making them ideal for emergency situations or responding to security incidents in a timely manner [92,93,95]. Rapid deployment allows for swift assessment and decision making by security personnel.

Crowd Monitoring. In crowded events, public gatherings, or protests, drones can be used to monitor crowd behavior, assess potential security risks, and ensure the safety of both the public and security personnel [88,94,95]. During major events, festivals, or gatherings, copter drones enhance event security by providing continuous aerial surveillance. They can monitor crowd movements, identify potential security threats, and assist in coordinating the response of security personnel.

Critical Infrastructure Inspection. Drones are employed to inspect critical infrastructure such as power plants, pipelines, and communication towers. They can provide visual inspections of hard-to-reach areas, helping to identify vulnerabilities or signs of unauthorized access [96].

Perimeter Security. Copter drones equipped with thermal imaging cameras can enhance perimeter security by detecting and monitoring movement in low-light conditions [89,90,92]. This capability is valuable for securing borders, critical installations, and industrial facilities. Drones are instrumental in monitoring remote or inaccessible areas, such as forests, coastlines, and mountainous regions. This capability is valuable for preventing illegal activities, including poaching, smuggling, and unauthorized access to protected areas.

Search and Rescue Operations. Drones assist in search and rescue missions by rapidly scanning large areas and identifying potential locations of interest [97]. They provide real-time data to rescue teams, optimizing their efforts and improving the chances of locating missing persons or responding to emergencies.

Traffic Management. Drones contribute to traffic management and accident response by providing real-time aerial views of roadways [88]. They assist authorities in assessing traffic conditions, monitoring accidents, and coordinating emergency responses.

Deterrence and Visibility. The presence of drones can act as a deterrent to potential security threats [98]. Knowing that an area is under drone surveillance can discourage criminal activities, enhancing overall security.

Data Analysis and Integration. Data collected by copter drones can be integrated with advanced analytics and artificial intelligence for more effective threat detection and pattern recognition [89,90,91,92,93,94]. This aids in proactive security measures and decision making.

In summary, copter drones play a crucial role in surveillance and security by offering a cost-effective, flexible, and rapidly deployable platform for monitoring and securing various environments. Their capabilities contribute to improved situational awareness, enhanced response times, and the overall effectiveness of security operations.

### 4.3. Agriculture

Drones have emerged as powerful tools in precision agriculture, revolutionizing the way that farmers monitor, manage, and optimize their crops. The integration of drone technology in agriculture provides farmers with valuable data for more informed decision making, resource optimization, and increased crop yields. What follows is an exploration of the applications of drones in precision agriculture [99,100,101,102,103,104].

Crop Monitoring and Health Assessment. Drones equipped with high-resolution cameras and sensors capture detailed images of crops [105]. These images help to assess the health and vigor of plants by identifying areas with pest infestations, diseases, nutrient deficiencies, or water stress. Early detection allows for targeted interventions, reducing the impact on crop yield.

Aerial Mapping and Surveying. Drones generate accurate and up-to-date aerial maps of fields [104,106]. This mapping assists farmers in assessing the topography, soil composition, and drainage patterns. By having a comprehensive understanding of their fields, farmers can optimize planting patterns, irrigation, and drainage systems.

Precision Planting. Drones equipped with precision seed dispensers can precisely plant seeds in predefined locations. This ensures optimal spacing between plants, leading to improved crop uniformity and resource utilization. Precision planting contributes to higher crop yields and resource efficiency [106,107].

Variable Rate Application. Drones enable a variable rate application of inputs such as fertilizers, pesticides, and herbicides [108]. By analyzing data from crop health assessments, drones can create prescription maps, guiding automated systems to apply inputs at variable rates across the field. This targeted approach minimizes input waste and reduces environmental impact.

Irrigation Management. Thermal imaging sensors on drones can identify variations in soil moisture levels [109]. This information assists farmers in optimizing irrigation schedules and identifying areas with overwatering or insufficient moisture. Improved irrigation management leads to water conservation and increased crop productivity.

Crop Scouting and Surveillance. Drones provide a quick and efficient means of crop scouting [102]. Farmers can regularly survey large areas, identifying potential issues and making timely decisions. Surveillance capabilities also aid in monitoring the growth stages of crops, allowing for precise management practices.

Livestock Monitoring. Drones are used to monitor and manage livestock, providing insights into herd movements, health, and behavior [110]. These data aid in optimizing grazing patterns, assessing the condition of animals, and improving overall livestock management.

Yield Prediction. Drones equipped with multispectral sensors capture data related to plant health and biomass [111]. By analyzing these data, farmers can make accurate predictions about crop yields. Yield prediction supports better planning for the harvesting, storage, and marketing of agricultural produce.

Disease and Pest Control. Drones equipped with sensors and cameras can detect signs of diseases and pest infestations early on [112,113]. This enables farmers to implement targeted and timely interventions, reducing the need for broad-spectrum chemical applications and minimizing the impact on beneficial organisms.

Data Integration and Analytics. Data collected by drones are integrated with advanced analytics and farm management software [114,115]. This integration allows farmers to make data-driven decisions, optimize inputs, and continually improve farming practices for sustainable agriculture.

In summary, drones play a crucial role in precision agriculture by providing farmers with accurate and timely information to enhance decision-making processes. From crop monitoring to variable rate application, drones contribute to increased efficiency, resource optimization, and sustainable agricultural practices.

### 4.4. Search and Rescue

Drones have become invaluable tools in search and rescue (SAR) operations, providing rapid and efficient assistance in locating missing persons, assessing disaster-stricken areas, and improving overall response capabilities. Below, a discussion on how drones are employed in search and rescue operations is presented [116,117,118,119].

Rapid Deployment. Drones offer quick and easy deployment, enabling search and rescue teams to cover large areas in a short amount of time [90,91,94]. Their ability to launch rapidly provides a crucial advantage in the early stages of a search operation.

Aerial Surveillance. Equipped with high-resolution cameras and thermal imaging sensors, drones provide a bird’s-eye view of the search area [120,121]. Aerial surveillance enhances the visibility of the terrain, making it easier to spot individuals or objects, especially in challenging or remote locations.

Night Vision Capabilities. Drones equipped with infrared or thermal cameras offer night vision capabilities [122,123]. This is particularly useful in search and rescue missions conducted during low-light conditions, allowing teams to locate individuals or detect heat signatures in darkness.

Terrain Mapping. Drones can create detailed maps of the search area, including topography and vegetation [124,125]. This information assists search and rescue teams in planning their approach, identifying potential obstacles, and optimizing routes to reach target locations more efficiently.

Communication Support. Drones equipped with communication equipment can serve as mobile communication hubs, helping to establish or strengthen communication links in areas with limited or disrupted communication infrastructure [126]. This is crucial for coordinating rescue efforts and relaying information to command centers.

Swift Survey of Disaster Zones. In the aftermath of natural disasters or emergencies, drones can swiftly survey disaster zones, assessing the extent of damage and identifying areas where rescue efforts are needed most urgently [127,128,129]. This information guides rescue teams in prioritizing their response.

Monitoring Hazardous Environments. Drones are capable of entering hazardous environments, such as collapsed buildings, unstable terrains, or areas with potential chemical hazards, without risking human lives [130,131]. They provide real-time visual information to assess the situation and plan safe and effective rescue operations.

Search Efficiency. Drones equipped with intelligent software and algorithms can autonomously search designated areas based on predefined patterns [132,133]. This automation enhances search efficiency, covering more ground in less time and improving the chances of locating missing persons or survivors.

Payload Delivery. Drones can be equipped with payload delivery systems to drop essential supplies, medical kits, or communication devices to individuals in distress [132,134]. This capability is particularly beneficial in situations where direct access by rescue teams is challenging.

Collaborative Swarms. Collaborative drone swarms, including both aerial and terrestrial unmanned vehicles, can be deployed to search large areas simultaneously [135,136,137,138,139]. These coordinated efforts increase the likelihood of finding missing persons or detecting signs of life in expansive search zones.

Post-Event Documentation. Drones assist in documenting the post-event scenario, capturing images and videos that aid in post-mission analysis, debriefing, and improving future search and rescue strategies [140,141].

In summary, drones play a vital role in search and rescue operations by providing a versatile, efficient, and technologically advanced means of surveying, locating, and assisting individuals in distress. Their capabilities contribute to faster response times, enhanced situational awareness, and improved overall effectiveness in search and rescue missions.

## 5. Drone Control Methods and Systems

**Figure 7:**
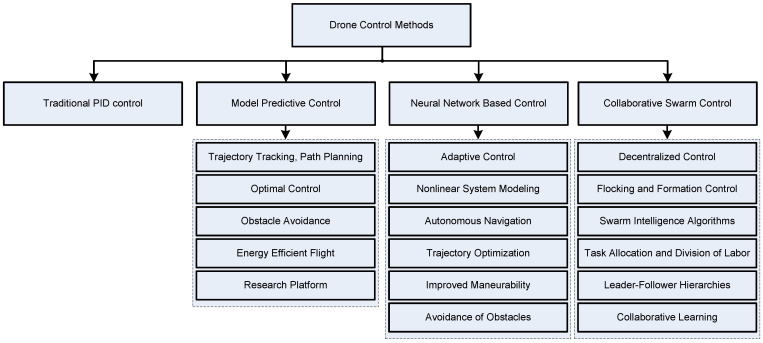
Drone control methods.

This section starts with an overview of typical electronic components used in drones, which is followed by a discussion on the most popular methods used to control drones, starting with the simplest traditional methods and closing with more advanced and sophisticated methods used to control drone swarms. Reviewed control methods are presented in Figure 7.

### 5.1. Typical Set of UAV Electronic Components

The electronic component of the UAV includes at least the following [142,143,144,145]:

- Flight controller;
- Accumulator;
- Brushless motor and ESC;
- Radio transmitters;
- Location and navigation sensors: GPS, air speed, and others;
- Video system (analog or digital): transmitter, receiver, camera, antenna.

The main part of the drone control system is a flight controller—an electronic device that controls the flight of an aircraft. The term is used for unmanned aerial vehicles, including aircraft models and drones. Usually, the flight controller receives commands from the radio control system, but it can work completely autonomously according to a predetermined flight plan or in the object tracing mode. Moreover, the autonomous flight mode is found both in personal “selfie-copters” and in advanced military devices.

The functions of the flight controller include the following [142,143]:

- Stabilization of the device in the air using sensors such as a gyroscope, accelerometer, compass (they are usually located on the flight controller board);
- Altitude maintenance using a barometric altimeter (the barometer is usually built into the flight controller) or using a GPS sensor;
- Heading speed measurement using a differential flight speed sensor (Pitot tube) or using a GPS sensor;
- Automatic flight to predetermined points (mission planner);
- Transmission of current flight parameters to the control panel;
- Ensuring flight safety (return to the take-off point in case of signal loss, automatic landing, automatic take-off);
- Stopping in front of an obstacle (for multicopters) or flying around obstacles (for airplanes) if sensors are available;
- Connection of additional peripherals: OSD (on-screen display), servo drives, LED indication, relay, and others.

The connection of third-party devices, such as a GPS, video transmitter, camera, differential speed sensor, etc., occurs through their ports, which are marked on the flight controller board and typically include the following [142,143]:

- UART (RX/TX)—universal asynchronous receiver–transmitter;
- USART (RX/TX)—universal synchronous–asynchronous receiver–transmitter;
- I2C (DA/CL or SDA/SCL)—inter-integrated circuit;
- SBUS—SPARC bus;
- CANBUS (RX/TX)—controller area network bus;
- VTX—video transmitter.

Flight controllers may implement different control principles, and the most popular, such as PID controllers, model predictive controllers, and neural-network-based controllers, are described in more detail in the following sub-sections.

### 5.2. PID Controllers

Proportional–integral–derivative (PID) controllers are widely used in the field of drone flight control systems to achieve stable and responsive performance. These controllers utilize three components—proportional (P), integral (I), and derivative (D)—to adjust the drone’s behavior and maintain a desired state [146,147].

The proportional component is responsible for correcting the current error in the system. Error, in the context of a PID controller, is the difference between the desired state (setpoint) and the actual state (output). The proportional term contributes to the correction by applying a force proportional to the current error. In drone flight control, the proportional term adjusts the drone’s response based on how far it is from its intended position. For example, if the drone is deviating from its desired altitude, the proportional term applies a force to bring it back to the setpoint [146,147,148,149,150].

The integral component addresses accumulated error over time. It adds up the errors that have occurred over time and adjusts the control signal to eliminate any persistent deviation from the setpoint. In drone flight control, the integral term helps to correct for steady-state errors that might arise due to external disturbances, wind, or imperfections in the drone’s dynamics. It contributes to ensuring that the drone reaches and maintains the desired state over the long term.

The derivative component anticipates future errors by considering the rate of change in the error. It dampens the system’s response by applying a force proportional to the rate at which the error is changing. In drone flight control, the derivative term helps to prevent overshooting and oscillations. If the drone is approaching the setpoint too quickly, the derivative term acts to slow down the rate of change, providing smoother and more controlled movements.

**Figure 8:**
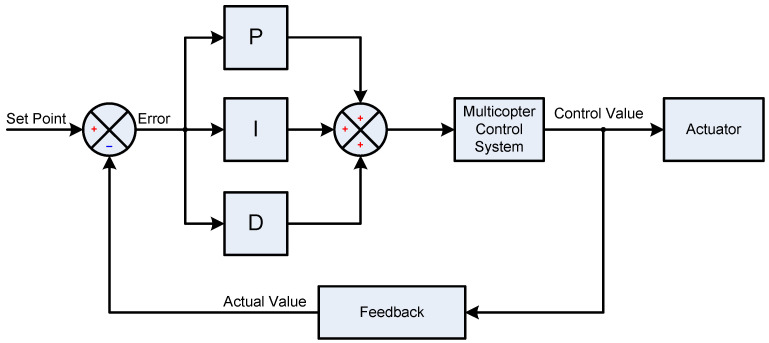
General principle of PID controller operation.

The PID controller combines the proportional, integral, and derivative components to calculate the control signal that adjusts the drone’s actuators (motors, propellers, etc.). The combined action aims to provide a balance between a fast response, minimal steady-state error, and reduced overshooting or oscillations. The general PID controller operation principle is shown in Figure 8.

The performance of a PID controller is highly dependent on tuning its parameters—proportional gain (Kp), integral gain (Ki), and derivative gain (Kd). Proper tuning is essential for achieving optimal stability, responsiveness, and robustness in the control system.

In conclusion, PID controllers in drone flight use the principles of proportional, integral, and derivative components to achieve stable and precise control. By adjusting these components and their associated gains, the controller can be tuned to meet the specific requirements of different drone models and environmental conditions, ensuring effective and reliable flight control.

### 5.3. Model Predictive Control (MPC)

Model predictive control (MPC) is an advanced control strategy that has gained popularity in drone flight control systems for its ability to handle complex dynamics, constraints, and uncertainties. MPC differs from traditional control methods by considering predictions of the system’s future behavior over a finite time horizon, optimizing control inputs to achieve specific objectives [151,152,153,154,155,156]. What follows is an exploration of the use of MPC for more advanced drone flight control strategies.

MPC requires an accurate dynamic model of the drone system, including its kinematics and dynamics. This model incorporates information about the drone’s structure, propulsion system, sensors, and environmental factors. Dynamic modeling is crucial for predicting the drone’s future states and responses.

This control type considers a prediction horizon, which defines the time span over which the system’s future states are predicted. Additionally, it employs a control horizon, which determines the time span over which control inputs are optimized [152]. Adjusting these horizons allows for a balance between accuracy and computational efficiency.

MPC is well suited for handling constraints in the system, such as physical limitations of the drone, safety constraints, and environmental restrictions [152,153,154]. The controller optimizes the control inputs while ensuring that the predicted future states satisfy these constraints, leading to safe and efficient operations.

Model predictive control is adaptable to changes in the operating conditions, disturbances, and uncertainties. It continuously updates predictions based on real-time sensor feedback, enabling the controller to respond to dynamic environmental conditions or unexpected events during flight [154,155].

This method can be used for trajectory tracking and path planning [155,156]. By optimizing the control inputs over a predictive horizon, MPC allows the drone to follow complex trajectories and paths accurately. This is particularly useful in applications like inspection, surveillance, and mapping, where precise movement is required.

**Figure 9:**
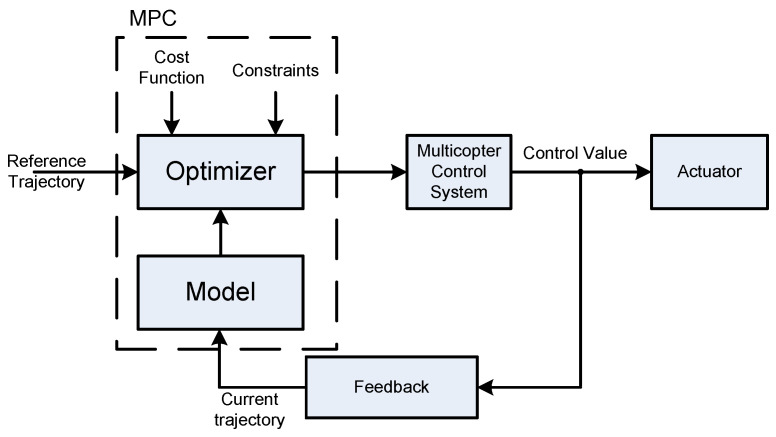
Simplified MPC operation principle.

MPC calculates the optimal control inputs by solving an optimization problem iteratively over the prediction horizon [151,155]. This allows the controller to adaptively adjust the control inputs based on the evolving system states, ensuring optimal performance. The simplified MPC principle is shown in Figure 9.

Also, MPC can be employed for obstacle avoidance by integrating obstacle information into the predictive model. The controller adjusts the drone’s trajectory to navigate around obstacles while respecting constraints and optimizing the overall flight path.

The use of MPC can contribute to energy-efficient drone flight. By considering the energy consumption model in the optimization process, the controller can minimize energy usage while meeting the mission objectives.

MPC allows for the simultaneous optimization of multiple objectives, such as reaching a destination quickly while minimizing energy consumption. This flexibility makes it suitable for applications where various criteria need to be considered simultaneously.

Finally, MPC provides a platform for researchers and engineers to experiment with and develop advanced control strategies. Its flexibility and adaptability make it a valuable tool for exploring innovative drone control approaches and addressing new challenges in the field.

Thus, MPC offers a powerful and flexible framework for implementing advanced drone flight control strategies. By considering predictive models, handling constraints, and optimizing control inputs over time, MPC enhances the capabilities of drones in terms of precision, adaptability, and efficiency in various applications.

### 5.4. Neural-Network-Based Control

Neural-network-based flight control has gained attention in recent years as a promising approach to enhancing the capabilities of copter drones. Neural networks, a subset of artificial intelligence (AI), offer the ability to learn complex relationships and patterns from data, allowing for adaptive and dynamic control strategies [157,158,159,160,161,162]. What follows is a discussion on the application of neural networks in enhancing flight control for copter drones.

Adaptive Control. Neural networks are capable of learning from data and adapting their behavior to varying flight conditions [163,164]. This adaptability is particularly valuable in dynamic environments where traditional control methods may struggle to provide optimal performance.

Nonlinear System Modeling. Copter dynamics are inherently nonlinear, and traditional control methods might face challenges in accurately modeling and controlling these systems [165]. Neural networks excel at capturing nonlinear relationships, making them well suited for modeling and controlling complex drone dynamics.

Sensor Fusion and Perception. Neural networks can integrate data from various sensors, such as accelerometers, gyroscopes, cameras, and GPS, to enhance perception and situational awareness [166]. This enables the drone to make more informed decisions based on a comprehensive understanding of its environment.

Learning and Optimization of Control Policies. Neural networks can be employed to optimize control policies, determining the best actions to take in different situations [167]. This is particularly beneficial for copter drones engaged in tasks like precision agriculture, where optimal trajectories and control strategies contribute to improved efficiency.

Autonomous Navigation. Neural networks enable copter drones to navigate autonomously by learning mapping and path planning functions [168]. This autonomy is valuable in applications such as search and rescue, surveillance, and exploration, where drones need to operate in challenging and dynamic environments.

Fault Tolerance. Neural-network-based flight control systems can exhibit a degree of fault tolerance [159]. By learning from diverse scenarios during training, the network may be more resilient to unexpected disturbances, sensor failures, or other issues that could arise during flight.

Trajectory Optimization. Neural networks can optimize trajectories for copter drones by learning patterns from historical flight data [169]. This is useful in applications like aerial photography or surveillance, where specific trajectories need to be followed for optimal data collection.

Real-time Adaptation. Neural networks can adapt in real time to changes in the environment or system dynamics [170]. This real-time adaptation is crucial for drones operating in unpredictable conditions, enabling them to maintain stability and responsiveness.

Improved Maneuverability. Neural-network-based controllers can enhance the maneuverability of copter drones, allowing for more agile and precise movements [171]. This is beneficial in applications such as drone racing or dynamic inspections where responsiveness is critical.

Avoidance of Obstacles. Neural networks can be trained to recognize and avoid obstacles during flight [172]. This obstacle avoidance capability enhances the safety of copter drones, especially in environments with complex structures or moving objects.

In summary, the application of neural networks in copter flight control holds great potential for enhancing adaptability, autonomy, and overall performance. From learning control policies to optimizing trajectories and improving fault tolerance, neural-network-based approaches contribute to advancing the capabilities of copter drones across a variety of applications.

### 5.5. Collaborative Swarm Control Strategies

These control types involve coordinating the actions of multiple drones to achieve collective goals efficiently and effectively. These strategies leverage principles from swarm intelligence, distributed control, and cooperation among agents. Novel algorithms, such as those described in [173,174], could be applied for this task. Typical collaborative swarm control strategies for drone systems include the following:

- Decentralized control, assuming distributed decision-making authority among individual drones, allowing them to make local decisions based on local information while coordinating with neighboring drones [175]. Each drone operates autonomously, reacting to its environment and communicating with nearby drones to achieve collective objectives without central coordination;
- Flocking and formation control, aiming to maintain desired spatial arrangements among drones, such as maintaining a formation shape or flying in a coordinated flock [176]. Drones adjust their positions and velocities based on local interactions with neighboring drones, following simple rules inspired by natural flocking behaviors observed in birds and insects;
- Swarm intelligence algorithms, such as ant colony optimization, particle swarm optimization, and artificial bee colony optimization, can be adapted for drone swarm control [177]. These algorithms enable drones to collectively explore, search, or optimize objectives in a distributed manner by sharing information and iteratively updating their behaviors;
- Task allocation and division of labor, which assign specific tasks or roles to individual drones based on their capabilities, resources, and proximity to the task [178]. Drones collaborate to divide complex tasks into smaller subtasks and allocate them among the swarm members, optimizing resource utilization and task efficiency;
- Leader–follower hierarchies, which establish hierarchical relationships among drones, with designated leaders guiding the behavior of follower drones [179]. Leaders may provide high-level commands or waypoints for followers to follow, while followers adjust their positions and velocities to maintain the formation relative to the leader;
- Collaborative learning and adaptation mechanisms, which enable drone swarms to learn from collective experiences, share knowledge, and adapt their behaviors over time [180]. Drones may employ machine learning algorithms, reinforcement learning, or evolutionary algorithms to improve performance, optimize strategies, and adapt to evolving mission scenarios.

By employing these collaborative swarm control strategies, drone systems can achieve enhanced coordination, efficiency, scalability, and robustness in various applications, including surveillance, search and rescue, environmental monitoring, infrastructure inspection, and delivery services.

## 6. Sensors and Perception

This section briefly discusses a typical set of sensors used in drones to ensure their operability, stability, and reliability during the flight.

### 6.1. Inertial Measurement Unit (IMU)

An inertial measurement unit (IMU) is a crucial component in a drone’s sensor suite, providing essential information about the drone’s orientation and acceleration. Comprising a combination of accelerometers and gyroscopes, an IMU works together to measure and report the drone’s motion in three-dimensional space. It includes the following components and features [181,182,183,184,185,186,187].

Accelerometers. Accelerometers within the IMU measure linear acceleration along the three axes (X, Y, and Z) in the drone’s body frame [185,187]. They detect changes in velocity and provide information about the drone’s linear motion. The accelerometers respond to both the drone’s acceleration due to external forces (such as wind or thrust changes) and the gravitational acceleration.

Gyroscopes. Gyroscopes in the IMU measure the drone’s angular rate of rotation around each axis [185,187]. They provide information about the drone’s rate of change in orientation, allowing for the calculation of the drone’s angular velocity. Gyroscopes are crucial for determining the drone’s rotational movements and changes in heading.

Integration for Orientation. By integrating the data from accelerometers over time, the IMU can determine the drone’s velocity [181,182,183]. Further integration of velocity provides information about the drone’s displacement or position. However, due to integration errors accumulating over time, the IMU alone is not sufficient for precise position estimation. Additional sensor data, such as those from a GPS or visual odometry, are often used to mitigate these errors.

Quaternion Representation. IMUs commonly provide orientation information in the form of quaternions [188]. Quaternions are a mathematical representation of orientation that avoids the issues associated with traditional Euler angles (yaw, pitch, and roll), such as gimbal lock. The quaternion representation ensures the accurate and continuous tracking of the drone’s orientation.

Attitude Stabilization. The IMU’s data on angular rates and accelerations are crucial for the drone’s flight controller to stabilize and control its attitude (orientation) [189]. By measuring deviations from the desired orientation, the flight controller adjusts the drone’s motor speeds to maintain stable flight.

Filtering Techniques. IMU data are susceptible to noise and drift over time [190]. Filtering techniques, such as the complementary filter or the Kalman filter, are commonly employed to improve the accuracy and reliability of the orientation estimates by combining IMU data with other sensor inputs.

Inertial measurement units are integral components of inertial navigation systems (INSs). In combination with other sensors like GPS, magnetometers, and barometers, IMUs contribute to precise navigation by continuously updating the drone’s position, velocity, and orientation.

Control Feedback Loop. IMU data are used in a closed-loop feedback system, providing real-time information to the flight controller [186]. The flight controller processes these data to make rapid adjustments to the drone’s motor outputs, ensuring stable flight and responsive control.

Low-Level Stabilization. IMUs are responsible for low-level stabilization tasks, including maintaining the drone’s level orientation, compensating for external disturbances, and enabling smooth transitions between different flight modes [191].

Calibration and Alignment. Proper calibration and alignment of the IMU are crucial for ensuring accurate sensor measurements [192]. Calibration procedures account for sensor biases, offsets, and misalignments, improving the overall reliability of the IMU data.

In summary, the inertial measurement unit plays a central role in providing critical information about a drone’s orientation and acceleration. These data are fundamental for flight control, stabilization, navigation, and overall performance in various operational scenarios. The integration of IMU data with other sensor inputs enhances the drone’s ability to navigate and operate effectively in diverse environments.

### 6.2. Global Positioning System and Global Navigation Satellite System (GPS and GNSS)

The Global Positioning System (GPS) and Global Navigation Satellite System (GNSS) are satellite-based navigation systems that play a crucial role in drone navigation, providing accurate position, velocity, and timing information [193,194]. The main GNSSs used in multirotor drones are the following [195]:

- GPS (Global Positioning System), which is the most common and best known GNSS. It consists of a satellite constellation that provides signals to determine the exact geographical position;
- GLONASS (Global Navigation Satellite System), which is the Russian alternative to the American GPS. It consists of a constellation of Russian satellites that provide navigation signals;
- Galileo, which is the European Union’s navigation system. It provides independent signals for navigation and positioning;
- BeiDou, which is the Chinese navigation system. It provides signals for navigation in China and neighboring regions;
- NavIC (Navigation with Indian Constellation), which is the Indian navigation system developed by the Indian Space Research Organization (ISRO).

Most modern multicopters are equipped with GNSS receivers that can work with different systems at the same time (e.g., GPS and GLONASS) to provide more accurate navigation. The GNSS is important for many aspects of multicopter flight, including stabilization, homing, pinpointing, and other functions.

The GPS and GNSS are used for drone navigation, such as for the following purposes [195].

Position Determination. The primary function of the GPS/GNSS for drones is to determine their precise location in three-dimensional space. By receiving signals from multiple satellites, the drone’s GPS/GNSS receiver calculates its position using trilateration, allowing for accurate latitude, longitude, and altitude coordinates.

Velocity Estimation. The GPS/GNSS enables drones to determine their speed and direction by analyzing changes in position over time. This velocity information is crucial for planning and executing flight maneuvers, adjusting the drone’s trajectory, and ensuring precise navigation.

Navigation and Waypoint Following. The GPS/GNSS is integral to drone navigation, especially in waypoint-based flight modes. Operators can define waypoints on a map, and the drone autonomously follows these waypoints using GPS coordinates. This feature is valuable for various applications, including aerial surveying, mapping, and surveillance.

Autonomous Flight. Drones leverage the GPS/GNSS for autonomous flight operations, enabling them to operate without continuous human intervention [196]. Autonomously controlled drones use GPS data to navigate, follow predetermined routes, and execute specific tasks, such as surveying large areas or inspecting infrastructure.

Return-to-Home (RTH) Functionality. The GPS/GNSS plays a critical role in the return-to-home feature, which allows drones to return to their take-off point automatically [197]. This feature is activated in scenarios such as low battery levels or loss of communication with the remote controller, ensuring that the drone returns safely.

Geofencing. The GPS/GNSS enables the implementation of geofencing, defining virtual boundaries within which the drone is allowed to operate [198]. This is crucial for compliance with regulations and preventing the drone from entering restricted or unsafe areas.

Precision Agriculture. In agriculture, drones equipped with a GPS/GNSS are used for precision farming [199]. Drones can follow precise routes for crop monitoring, imaging, and application of fertilizers or pesticides, optimizing resource usage and improving crop yields.

Mapping and Surveying. The GPS/GNSS enhances the accuracy of mapping and surveying tasks performed by drones [200]. The precise positioning information allows for the creation of detailed and georeferenced maps, topographic surveys, and 3D models.

Real-Time Kinematic (RTK) GPS. The RTK GPS is an advanced GPS/GNSS technique that uses correction data from a ground station to achieve centimeter-level accuracy [201]. Drones equipped with an RTK GPS are employed in applications requiring extremely high precision, such as land surveying and construction site monitoring.

Emergency Response and Search and Rescue. The GPS/GNSS is crucial for drones involved in emergency response and search and rescue operations [132]. Accurate positioning information aids in locating individuals or incidents, optimizing the deployment of resources, and improving overall operational efficiency.

Timing Synchronization. The GPS/GNSS provides accurate timing synchronization for drones [202]. This is essential for coordinating the actions of multiple drones in a swarm, facilitating collaborative tasks and ensuring precise timing in various applications.

Integration with Other Sensors. GPS/GNSS data are often integrated with information from other sensors, such as IMUs, barometers, and magnetometers, to enhance overall navigation accuracy, especially in scenarios where GPS signals may be temporarily obstructed or degraded [203].

So, the GPS and GNSS are fundamental to drone navigation, enabling accurate positioning, velocity estimation, and autonomous flight capabilities. Drones leverage these satellite-based systems across various industries, enhancing their operational capabilities and contributing to the development of innovative applications.

### 6.3. Computer Vision

The integration of computer vision for object detection and recognition in drone-based applications has become increasingly prevalent, opening up new possibilities and enhancing the capabilities of UAVs. Computer vision allows drones to autonomously process visual information, identify objects, and make informed decisions based on their surroundings [204,205,206,207,208,209]. This provides the following possibilities and features.

Object Detection. Computer vision enables drones to identify and locate objects within their field of view [204,206,207,208,209]. Object detection algorithms, such as YOLO (You Only Look Once) [210] or SSD (Single Shot Multibox Detector) [211], are commonly employed to detect and draw bounding boxes around objects in real time. This is useful in applications such as surveillance, search and rescue, and monitoring large areas.

Object Tracking. Object tracking algorithms in computer vision allow drones to follow and monitor the movement of identified objects over time [212]. This is beneficial for tracking moving targets, such as vehicles, individuals, or wildlife, and is applicable in scenarios like aerial cinematography, security surveillance, and wildlife monitoring.

Automated Inspection. Drones equipped with computer vision systems can autonomously inspect and identify structural defects, damages, or anomalies in infrastructure. This is valuable for applications in industries such as construction, energy, and telecommunications, where routine inspections are essential.

Precision Agriculture. In agriculture, computer vision on drones can identify and analyze crops, monitor plant health, and detect issues such as diseases or nutrient deficiencies [203,205,206,207,208,209]. This information aids farmers in making data-driven decisions for precision agriculture, optimizing crop management, and improving yields.

Environmental Monitoring. Drones with computer vision capabilities contribute to environmental monitoring by detecting and analyzing changes in ecosystems, wildlife habitats, and natural resources [213]. This is crucial for applications like biodiversity assessment, deforestation monitoring, and climate research.

Mapping and 3D Reconstruction. Computer vision facilitates the creation of high-resolution maps and 3D reconstructions by processing visual data captured by drones [214]. This is valuable in urban planning, construction site monitoring, and archaeological surveys, providing detailed and accurate representations of the terrain.

Object Recognition and Classification. Beyond detection, computer vision enables drones to recognize and classify objects based on their visual characteristics [215]. Deep learning models, such as convolutional neural networks (CNNs), are employed for tasks like identifying specific objects, animals, or structures. This is applicable in diverse fields, including wildlife conservation, public safety, and infrastructure inspection.

Augmented Reality (AR) Applications. Computer vision contributes to augmented reality experiences in drone applications [216]. By recognizing and overlaying digital information on real-world objects, drones can provide enhanced situational awareness or deliver interactive experiences for users in fields like tourism, education, or entertainment.

Event Security and Crowd Monitoring. Drones equipped with computer vision systems contribute to event security by monitoring crowds, detecting anomalies, and identifying potential security threats [95,96]. This enhances situational awareness during large gatherings, concerts, or public events.

Search and Rescue Operations. Computer vision aids in search and rescue missions by helping drones to identify and locate missing persons or objects in challenging environments [118,119,120,121]. It enhances the efficiency of search operations, especially in scenarios with dense vegetation, rough terrain, or low visibility.

Collision Avoidance. Computer vision systems contribute to collision avoidance by enabling drones to detect and react to obstacles in their flight path [217]. This is crucial for safe and autonomous navigation, preventing collisions with buildings, trees, or other drones.

Thus, the integration of computer vision for object detection and recognition significantly expands the capabilities of drones across various applications. By processing visual information in real time, drones equipped with computer vision systems can autonomously interpret their environment, make informed decisions, and execute tasks with a high degree of precision and efficiency.

### 6.4. Environmental Information (SLAM)

SLAM (simultaneous localization and mapping) is a technology that allows an unmanned aerial vehicle to simultaneously determine its position in space and create a map of the environment [218,219,220,221,222,223].

- The main components of SLAM for drones include the following:
- Cameras used to obtain visual data environment;
- Laser rangefinders (LiDARs), which can be used to measure distances and create accurate three-dimensional maps of the environment;
- Depth cameras that measure distances to objects and allow one to estimate the depth of objects in the image;
- Inertial sensors providing information on the acceleration and rotation of the drone;
- Data processing systems and SLAM algorithms that analyze input data and use them to determine the position and create a map.

SLAM is used to provide the following:

- Autonomous flight. SLAM allows the drone to navigate in an unknown environment, simultaneously creating a map and determining its position;
- Avoiding obstacles. This technology helps in avoiding collisions with obstacles, as the drone can detect objects and avoid them;
- Stabilization and accurate position maintenance. SLAM helps to keep the drone stable in the air even when there is no access to the GNSS signal;
- Environmental mapping. The system creates accurate three-dimensional maps of the environment that can be used for further analysis or navigation;
- Recognition and tracking of objects. The technology allows the drone to recognize and track the movement of objects around it.

SLAM is a very useful technology for drones, especially in environments where it is difficult or impossible to use a GNSS (for example, indoors or in areas with complex magnetic fields).

### 6.5. Short-Range Radio Navigation Systems (VOR, DME)

Short-range radio navigation systems (VOR—VHF omnidirectional range, DME—distance-measuring equipment) are used in aviation to navigate and determine distances between aircraft and ground points [224,225].

However, for multirotor drones, these systems are generally not standard equipment. Instead, drones usually specialize in using global navigation satellite systems such as a GPS, GLONASS, Galileo, etc.

The main reasons for why multirotor drones do not use VOR and DME are as follows:

- The equipment size and weight of VOR and DME require large and heavy antennas and equipment that is difficult and inconvenient to install on a drone;
- Frequency bands. VOR and DME operate in the high-frequency radio range, requiring large antennas and powerful transmitters;
- Licensing and regulation. The use of VOR and DME requires special permits and licenses from regulatory authorities;
- Intentional range limitations. VOR and DME are for aviation use and have a limited range that varies from airport to airport;
- Low compatibility with drones. The use of VOR and DME in multirotor drones may cause electromagnetic interference and affect the normal operation of other electronic components.

So, while VOR and DME are useful in aviation, they are not standard components for multirotor drones.

### 6.6. Object Detection and Tracking

Object detection and tracking are important functions for multirotor drones, as they allow one to automatically detect and track the movement of objects in the environment. For these tasks, various technologies and algorithms are used [226,227,228,229,230,231,232].

The main components of object recognition and tracking systems in multirotor drones include the following:

- Cameras and visual systems. Cameras, especially those with high resolution and high frame rates, are the primary source of visual input. They allow the drone to see its surroundings;
- Depth sensors, such as LiDAR or depth cameras, which provide additional information about distances to objects. This can be useful in recognizing and avoiding obstacles;
- Artificial intelligence (AI) and machine learning. These methods are used to train object recognition models. They can detect and classify objects in images;
- Tracking algorithms, which allow the drone to determine the path of movement and accurately track the movement of objects in time;
- Hybrid systems. Some solutions use a combination of cameras, LiDAR, and other sensors to obtain a more complete picture of the environment.

The application of these technologies can be useful for multirotor drones in many scenarios, including tracking objects to create dynamic images, navigating around obstacles, creating 3D models, and more.

However, it is important to remember that the efficient operation of these systems requires powerful computing resources and appropriate data-processing algorithms.

## 7. UAVs Software

The typical software used to configure and control multirotor drones is as follows [233,234,235]:

- Ardupilot + Mission Planner;
- Betaflight;
- INAV;
- Qground Control + PX4.

### 7.1. Ardupilot and Mission Planner

Ardupilot is an open-source autopilot software designed to control UAVs and other autonomous systems [236,237,238,239].

It provides a number of functions and capabilities for UAV navigation and control.

Here are some key features of Ardupilot:

- Multi-platform. Ardupilot supports various operating systems, including Linux, Windows, and MacOS;
- Versatility. It supports a wide range of different types of drones, including quadcopters, airplanes, helicopters, gliders, and more;
- Automated missions. Ardupilot provides the ability to create and execute automated missions, including point, line, circular trajectories, and more;
- Different flight modes, including Loiter (maintain position), RTL (return to launch), Guided (piloting using points on the map), and others;
- Open-source, allowing the development community to adapt and modify the software.

Mission Planner is a software for controlling and configuring Ardupilot autopilots. It provides an interface to interact with Ardupilot and control various parameters and functions [240,241]. Here are some key features of Mission Planner:

- Parameter control. It allows users to configure various autopilot parameters such as PID controllers, speed limits, geofences, and more;
- Mission creation, which provides tools to create automated missions with waypoints, actions, and conditions;
- Monitoring and diagnostics—a visual interface for monitoring data from the autopilot, including telemetry, logs, graphs, and more;
- Three-dimensional modeling, which allows one to display a three-dimensional model of the terrain and flight path;
- Integration with Google Earth, providing the ability to import and export mission data to Google Earth.

Mission Planner is a powerful tool for configuring and controlling Ardupilot-based drones. It is used to plan and execute various missions, as well as to configure flight modes and other parameters.

### 7.2. Betaflight

Betaflight is an open-source software specifically designed for FPV (first-person view) control of UAVs, specifically quadcopters [232,242,243].

Here are some key features and capabilities of Betaflight:

- Focus on FPV. Betaflight specializes in the most popular types of UAVs for FPV, including quadcopters;
- High speed and accuracy of control. The software is designed with the needs of racers and pilots in mind, who perform complex maneuvers and stunts;
- A wide range of PID settings allows one to fine-tune the control parameters of the flight platform for optimal performance and stability;
- Various flight modes, including Acro, Angle and Horizon;
- Automatic modes, including stabilization modes, RTL (return to launch), and others;
- Monitoring of the state of the flight platform. Betaflight provides tools for displaying and analyzing data from the flight platform, including telemetry, graphs, and more;
- Support for various hardware platforms. This software is compatible with many types of flight platform controllers;
- Open-source code that allows community developers to make changes and extend the capabilities of the software.

Betaflight is a popular choice among FPV racers and pilots who value precision and reliability in controlling their quadcopters.

### 7.3. INAV (Intelligent Navigation)

INAV is an open-source autopilot software designed to control fixed-wing UAVs such as airplanes, winged drones, and gliders. INAV provides an opportunity to automate and improve the navigation functions of these devices [244,245,246].

Here are some key features of INAV:

- Focus on airplanes and winged drones. INAV specializes in the control and navigation of aerodynamic UAVs, where aerodynamic control is essential;
- Stability and navigation. The software provides the ability to automatically control flight stability and navigation, including modes that allow one to maintain a stable position and perform automatic tasks;
- Automated missions. INAV enables the planning and execution of automated missions, including point missions, trajectories, and path tracking;
- Automatic take-off and automatic landing;
- Support for GPS and other sensors. INAV interacts with various sensors, including a GPS, compasses, and others for precise navigation and orientation;
- Open-source code. As open-source software, INAV allows the development community to make changes and develop additional functionality.

INAV is an effective software for automating and improving the navigation functions of aerodynamic UAVs. It is used in many scenarios, including aerial photography, research missions, and others.

### 7.4. Qground Control + PX4

QGroundControl is an open-source software used to control and configure UAVs and unmanned land or water vehicles [247,248,249,250]. Here are some key features of QGroundControl:

- Multi-platform. The software supports various operating systems, including Windows, macOS, and Linux;
- Open-source code. QGroundControl allows users to modify and adapt it to their own needs;
- Configuration and control. QGroundControl allows users to configure and control flight platform parameters, including flight modes, altitude, speed, pitch angles, and more;
- Missions and ways. It is possible to create and execute automatic missions, including point, line, and others;
- Monitoring and debugging. QGroundControl provides various tools for monitoring the state of the vehicle, including displaying telemetry, logs, graphs, etc.

PX4 is an open-source autopilot designed to control UAVs and other autonomous systems. It can be used on various types of UAVs, including quadcopters, airplanes, helicopters, and others [251,252,253].

Here are some key features of PX4:

- Versatility. PX4 is a versatile autopilot that can be used for various types of UAVs, including multicopters, gliders, helicopters, and more;
- Flight platform control algorithms. PX4 provides a wide range of control algorithms, allowing users to customize the parameters of the flight platform;
- Mission support and navigation. PX4 provides the ability to create and execute automatic missions using different navigation algorithms;
- Development environment (DevKit). It provides tools for developing and testing additional autopilot software;
- Open-source code. PX4 is based on open-source code, allowing users to adapt and modify it to their needs.

It is important to remember that PX4 is autopilot software, while QGroundControl is a UAV control and configuration program that can work with a variety of autopilots, including PX4.

### 7.5. Comparison of Ardupilot and PX4

Ardupilot and PX4 are two different platforms for controlling UAVs, each with its own characteristics and key features.

Let us start with the common features of both platforms:

- Versatility. Both platforms can be used to control various types of UAVs, including quadcopters, airplanes, helicopters, gliders, and more;
- Open-source code. Both Ardupilot and PX4 are based on open-source code, allowing the development community to make changes and extend the capabilities of the software.

Features of Ardupilot:

- Functionality. It provides a rich set of features, including multiple flight modes, automated missions, auto search and rescue, GPS support, remote control, and more;
- Community of developers. It has an active and large community of developers and users who contribute to the continuous improvement and support of the platform.

Features of PX4:

- Architecture and algorithms. PX4 uses different control and navigation algorithms that allow for high accuracy and reliability;
- Documentation and support. PX4 has detailed documentation and an active user community that helps beginners and advanced users to learn and use the platform.

Both platforms are powerful and excellent choices for drone control. Ardupilot may be a better choice for those who work with a wide range of drones and want to use software with a large developer community. PX4 will be a more versatile option for those looking for a multi-functional autopilot for various types of UAVs. Both of these options are high-quality and reliable, so the choice will depend on the specific needs and priorities of the user.

## 8. Challenges and Future Directions

### 8.1. Current Challenges

While copter drone technology has advanced rapidly in recent years, there are still several challenges and limitations that the industry faces. Some of the most important current challenges are listed below.

Limited Battery Life. Battery technology remains a significant limitation for copter drones [254]. The limited energy storage capacity constrains the flight time, making it challenging to perform extended missions or cover large areas without requiring frequent battery changes.

Payload Capacity. Copter drones, especially smaller consumer models, often have limited payload capacity [255]. This restricts the types of sensors, cameras, or equipment that can be carried, limiting the range of applications in fields such as surveying, mapping, and delivery.

Vulnerability to Weather Conditions. Adverse weather conditions, such as strong winds, rain, or snow, can significantly impact the performance of copter drones [256]. Weather-related challenges include reduced stability, decreased flight efficiency, and potential damage to sensitive components.

Autonomy and Obstacle Avoidance. While there have been advancements in autonomous flight capabilities, achieving reliable obstacle avoidance and navigation in complex environments remains a challenge [257]. Copter drones may struggle to detect and navigate around obstacles with high precision, posing risks during flight.

Regulatory and Legal Challenges. The regulatory environment for drone operations is continually evolving [18]. Compliance with airspace regulations, privacy concerns, and obtaining proper permits can be challenging. Harmonizing international regulations and addressing privacy issues are ongoing challenges for the drone industry.

Limited Range and Communication. Copter drones typically have a limited communication range, which can restrict their operational radius [258]. This limitation is especially relevant for applications that require long-range communication, such as remote sensing, agriculture, or monitoring large infrastructure projects.

Security and Counter-Drone Measures. Concerns related to the security of drone operations and the potential misuse of drones have led to the development of counter-drone technologies [259]. Securing drone communication and preventing unauthorized access or interference are challenges that need to be addressed.

Noise Pollution. Copter drones can generate significant noise, especially during take-off and landing [260]. Noise pollution can be a concern, particularly in urban environments or areas with noise-sensitive populations, impacting the social acceptance of drone technology.

Cost of Technology. High-quality sensors, advanced flight controllers, and other technological components can contribute to the overall cost of copter drones [261]. The expense may limit accessibility for certain applications or industries, hindering widespread adoption.

Integration with Air Traffic Management. Integrating drones into existing air traffic management systems presents challenges related to coordination, communication, and ensuring the safe coexistence of drones with manned aircraft [262]. Developing standardized procedures for drone traffic management is an ongoing effort.

Environmental Impact. The environmental impact of drone operations, including the carbon footprint associated with manufacturing and the potential for ecological disturbances during flight, is an area of concern [263]. Efforts to develop environmentally friendly drone technologies are needed.

Public Perception and Acceptance. Public acceptance of drone technology varies, and concerns about privacy, safety, and noise can influence public perception [264]. Effective public outreach and education are essential for addressing misconceptions and building trust in the use of copter drones.

Addressing these challenges will require ongoing collaboration between industry stakeholders, regulatory bodies, researchers, and technology developers to advance copter drone technology responsibly and sustainably.

### 8.2. Emerging Technologies

Copter drone technology continues to evolve, driven by ongoing research, technological innovations, and industry demands. Several emerging technologies and trends are shaping the future of copter drones, expanding their capabilities and enabling new applications, such as the following.

Longer Flight Times. Advances in battery technology, including a higher energy density and more efficient power management, are expected to lead to longer flight times for copter drones [265]. Extended endurance will enhance the feasibility of various applications, such as surveillance, mapping, and monitoring.

Hybrid Power Systems. Hybrid power systems, combining traditional batteries with alternative power sources like fuel cells or solar panels, are being explored to address the limitations of battery technology [266]. These systems aim to provide longer flight durations and an increased range for copter drones [267,268,269,270].

Swarming Technology. Swarming technology enables the coordination and collaboration of multiple drones to work together seamlessly [271]. This can enhance the efficiency of tasks such as search and rescue, environmental monitoring, and large-scale surveying. Swarm intelligence algorithms are being developed for decentralized control and communication among drone swarms.

Advanced Computer Vision. Continued advancements in computer vision algorithms and hardware are enhancing the object detection, tracking, and recognition capabilities of copter drones [272]. This technology will enable more sophisticated autonomous navigation, precision landing, and improved situational awareness in complex environments.

AI-Powered Navigation and Decision Making. The integration of artificial intelligence (AI) and machine learning (ML) algorithms into copter drone systems will enhance their ability to learn and adapt to changing environments [273]. AI-driven navigation and decision making will contribute to improved autonomy, allowing drones to handle complex tasks with minimal human intervention.

Edge Computing for Onboard Processing. Edge computing involves processing data locally on the drone rather than relying solely on remote servers. This can lead to faster data analysis, reduced latency, and improved real-time decision making, making copter drones more responsive and adaptable in dynamic situations [274,275,276].

Fifth-generation Connectivity. The deployment of 5G networks will provide copter drones with faster and more reliable communication capabilities [277]. Enhanced connectivity will support real-time data transmission, enabling applications like live streaming, remote sensing, and collaborative missions with minimal latency.

Foldable and Modular Designs. Advances in materials and design technologies are leading to more compact, foldable, and modular drone designs [278]. This trend facilitates easier transportation, storage, and customization, making copter drones more versatile for different applications and user preferences.

Sense-and-Avoid Systems. Sense-and-avoid systems are becoming increasingly sophisticated, incorporating a combination of sensors such as LiDAR, radar, and advanced computer vision. These systems enhance the ability of copter drones to detect and navigate around obstacles autonomously, improving overall safety and reliability.

Bio-Inspired Design. Bio-inspired design, drawing inspiration from nature, is influencing the development of more efficient and agile copter drones. Biomimicry in propulsion systems, wing designs, and overall aerodynamics can lead to improvements in efficiency, stability, and maneuverability [279,280,281].

Regulatory Frameworks and Traffic Management Systems. Advancements in regulatory frameworks and the development of more robust traffic management systems for drones will be crucial for safely integrating copter drones into airspace. Standardized procedures, automated air traffic control, and collaborative efforts between industry and regulators are expected to drive progress in this area.

Environmental Sustainability. Increasing emphasis on environmental sustainability is leading to the development of eco-friendly drone technologies [64]. This includes the use of biodegradable materials, energy-efficient components, and environmentally conscious manufacturing processes.

As these trends and technologies continue to mature, copter drones are likely to play an even more significant role in various industries, contributing to advancements in fields such as transportation, agriculture, public safety, and environmental monitoring. Ongoing research and collaboration within the drone industry will further drive the evolution of copter drone technology.

## 9. Conclusions

This paper reviews the current state of the art on copter drones and flight control systems. A general classification of unmanned aerial vehicles was provided, identifying multirotor drone systems as the most popular for individual use due to their relative simplicity and cheaper price compared to other UAV types. Types of the most popular multicopter systems, such as quadcopters, hexacopters, octocopters, and VTOL drones, as well as their main features, advantages, and disadvantages, were analyzed. The main application areas of copter drones, such as photography and videography, surveillance and security, agriculture, and search and rescue, were described. Also, typical control and electronic components used for drones were discussed, including different types of control methods, such as PID-controller-based, model predictive control, and neural-network-based control. A typical set of sensors used in drone building was overviewed as well, including inertial measurement units, global positioning systems and global navigation systems, simultaneous localization and mapping systems, computer vision tools, short-range radio navigation systems, and object detection and navigation systems. The most popular software used to control drones and plan their flight was presented, focusing on free-ware solutions. Finally, challenges and future directions in drone systems were discussed, leading to the conclusion that, as copter drone technology continues to advance, interdisciplinary research across engineering, computer science, environmental science, and social sciences will play a crucial role in shaping the future of drone applications. These research areas can contribute to the development of safer, more efficient, and socially responsible copter drone technologies.

## Figures and Tables

**Table 1:** UAV classification according to UVSI.

Group	Sub-Group	Flight Mass, kg	Flight Range, km	Max Flight Altitude, m	Flight Endurance, h
Small UAVs	Nano	<0.025	<1	100	<0.5
	Micro	<5	<10	250	1
	Mini	20–150	<30	150–300	<2
Tactic	Light UAVs for controlling the front edge of defense	25–150	10–30	3000	2–4
	Light Close-Range	50–250	30–70	3000	3–6
	Light Short-Range	150–500	70–200	5000	6–10
	Medium-Range	500–1500	>500	8000	10–18
	Medium-Range Endurance	250–2500	>250	50–9000	0.5–1
	Low-Altitude Deep Penetration	15–25	>500	3000	>24
	Low-Altitude Long Endurance	1000–5000	>500	5000–8000	24–48
	Medium-Altitude Long Endurance	2500–5000	>2000	20,000	24–48
Strategic	Combat UAVs (Shock)	>1000	1500	12,000	2
	UAVs equipped with a lethal warhead	150–1000	300	4000	3–4
	Decoy UAV	150–500	0–500	50–5000	<4
Special Purpose	Stratospheric UAVs	>2500	>2000	>20,000	>48
	Exo-stratospheric UAVs	>2500	>2000	>30,500	>48

**Table 2:** A comparison between quad-, hexa-, and octocopters and VTOLs.

Factor	Quadcopters	Hexacopters	Octocopters	Fixed-Wing VTOL Drones
Maneuverability	offer good maneuverability, with the ability to perform agile movements and hover in place. However, they may lack stability in windy conditions due to their fewer rotors	provide increased stability and redundancy compared to quadcopters, offering better maneuverability in adverse weather conditions and allowing for safer flights in case of motor failure	offer even greater stability and redundancy than hexacopters, making them suitable for more demanding applications such as heavy payload lifting, aerial cinematography, and industrial inspections	combine the vertical take-off and landing capabilities of copter drones with the efficiency and endurance of fixed-wing aircraft. While they may not be as agile as copter drones, they excel in covering large distances and conducting long-endurance missions
Payload Capacity	typically have a lower payload capacity compared to hexacopters and octocopters due to their fewer rotors and smaller size. They are suitable for carrying lightweight cameras and sensors	offer a higher payload capacity than quadcopters, making them suitable for carrying larger cameras, heavier sensors, and additional equipment	have the highest payload capacity among the three copter types, capable of lifting even heavier payloads such as professional cinema cameras, LiDAR systems, or specialized industrial equipment	typically have a higher payload capacity than copter drones, allowing them to carry larger payloads over longer distances. They are suitable for applications requiring heavy equipment or cargo transportation
Endurance	generally have shorter flight times compared to hexacopters, octocopters, and fixed-wing VTOL drones due to their higher power consumption and reliance on rotor-based propulsion	offer longer flight times than quadcopters due to their additional rotors and increased efficiency. They can typically fly for 20–30 min on a single battery charge	provide even longer flight times than hexacopters thanks to their additional redundancy and stability features. They can fly for 30 min to over an hour depending on the payload and operating conditions	offer the longest flight times among the compared platforms, with some models capable of flying for several hours on a single battery charge or tank of fuel
Speed and Range	typically have lower maximum speeds and shorter ranges compared to fixed-wing VTOL drones. They are best suited for short-range missions and tasks requiring precise maneuverability	can achieve higher speeds and cover longer distances than quadcopters, making them suitable for applications such as aerial photography, surveying, and mapping over larger areas	offer similar speed and range capabilities to hexacopters but with added redundancy and stability. They are suitable for more demanding missions requiring longer flight durations and higher payload capacities	excel in speed and range, capable of covering distances of tens or hundreds of kilometers in a single flight. They are ideal for long-range reconnaissance, mapping large areas, and delivering cargo over extended distances
Versality and Adaptability	suitable for a wide range of applications, such as aerial photography, videography, inspections, and recreational flying. They are easy to transport and operate in confined spaces	offer enhanced versatility and adaptability compared to quadcopters, with improved stability and payload capacity. They are used in applications requiring higher performance and reliability	provide the highest level of versatility and adaptability among the copter drones, capable of handling demanding tasks such as heavy lifting, industrial inspections, and aerial cinematography in challenging environments	combine the versatility of copter drones with the efficiency and endurance of fixed-wing aircraft, offering adaptability for a wide range of missions, including mapping, surveying, surveillance, and cargo delivery in both urban and remote areas

## Data Availability

Not applicable.
